# The Exposure to Different Photoperiods Strongly Modulates the Glucose and Lipid Metabolisms of Normoweight Fischer 344 Rats

**DOI:** 10.3389/fphys.2018.00416

**Published:** 2018-04-19

**Authors:** Roger Mariné-Casadó, Cristina Domenech-Coca, Josep M. del Bas, Cinta Bladé, Lluís Arola, Antoni Caimari

**Affiliations:** ^1^Technological Unit of Nutrition and Health, Eurecat, Technology Centre of Catalonia, Reus, Spain; ^2^Nutrigenomics Research Group, Department of Biochemistry and Biotechnology, Universitat Rovira i Virgili, Tarragona, Spain

**Keywords:** photoperiod, circannual rhythms, insulin sensitivity, glucose metabolism, lipid metabolism

## Abstract

Seasonal variations in day length trigger clear changes in the behavior, growth, food intake, and reproductive status of photoperiod-sensitive animals, such as Fischer 344 rats. However, there is little information about the effects of seasonal fluctuations in day length on glucose and lipid metabolisms and their underlying mechanisms in this model. To gain knowledge on these issues, three groups of male Fischer 344 rats were fed with a standard diet and exposed to different photoperiods for 14 weeks: normal photoperiod (L12, 12 h light/day), long photoperiod (L18, 18 h light/day), and short photoperiod (L6, 6 h light/day). A multivariate analysis carried out with 239 biometric, serum, hepatic and skeletal muscle parameters revealed a clear separation among the three groups. Compared with L12 rats, L6 animals displayed a marked alteration of glucose homeostasis and fatty acid uptake and oxidation, which were evidenced by the following observations: (1) increased circulating levels of glucose and non-esterified fatty acids; (2) a sharp down-regulation of the phosphorylated Akt2 levels, a downstream post-receptor target of insulin, in both the soleus and gastrocnemius muscles; (3) decreased expression in the soleus muscle of the glucose metabolism-related microRNA-194 and lower mRNA levels of the genes involved in glucose metabolism (*Irs1*, soleus, and *Glut2*, liver), β-oxidation (*Had* and *Cpt1*β, soleus) and fatty acid transport (*Cd36*, soleus, and liver). L18 animals also displayed higher blood glucose levels than L12 rats and profound changes in other glucose and lipid metabolism-related parameters in the blood, liver, and skeletal muscles. However, the mechanisms that account for the observed effects were less evident than those reported in L6 animals. In conclusion, exposure to different photoperiods strongly modulated glucose and lipid metabolisms in normoweight rats. These findings emphasize the relevance of circannual rhythms in metabolic homeostasis regulation and suggest that Fischer 344 rats are a promising animal model with which to study glucose- and lipid-related pathologies that are influenced by seasonal variations, such as obesity, cardiovascular disease and seasonal affective disorder.

## Introduction

It has generally been established that many mammals are season- or photoperiod-sensitive and are able to change their behavior, morphology and physiology to anticipate climate and food availability changes among the seasons (Prendergast, 2005; Morgan et al., 2006). Thus, food intake, growth, energy balance and reproduction have been observed to vary during the year in some species to ensure their survival (Ebling, 2015). This seasonal responsiveness can be regulated by two mechanisms: the first is promoted by environmental cues that indicate the time of year, such as the day length (photoperiod), in which melatonin plays a key role (Nelson and Demas, 1997); the second corresponds to the endogenous circannual rhythms, which are adjusted by environmental cues and can adapt to seasonal processes as a response to variations in the photoperiod or other external signals (Paul et al., 2008). Despite the limited impact of seasonality because of the increased use of artificial lighting, heating and air conditioning systems, humans also display seasonal changes in different anthropometric, physiologic, metabolic and behavior parameters (Pell and Cobbe, 1999; Reilly and Peiser, 2006; Haus, 2007; Kanikowska et al., 2014). Thus, the body fat significantly increases during winter in latitudes far from the equator, where greater variations in temperature, climate and daylight hours are registered, and the levels of physical activity and energy expenditure are lower in the winter than in the summer (Reilly and Peiser, 2006; Kanikowska et al., 2014). Since fat accretion and decreased physical activity can increase the risk of insulin resistance and cardiovascular disease (CVD), the seasonal variations in these and other parameters can place the human health at more risk in winter than in summer (Reilly and Peiser, 2006). This fact is illustrated by the increased number of cardiac events observed in winter both north and south of the equator, by the additional 20,000 deaths per year caused by coronary and cerebrovascular events that were reported in England and Wales during this season, and by the strong negative correlation found between CVD mortality and the hours of sunshine (Pell and Cobbe, 1999). However, since the seasonal variation of these risk factors that trigger a peak winter mortality is influenced by changes in other exogenous factors different than day length, such as temperature and lifestyle (Pell and Cobbe, 1999; Reilly and Peiser, 2006; Kanikowska et al., 2014), the relevance of seasonal variations in day length on health is far from being established. In this sense, the use of animal models, which can be maintained under constant temperature and social input, have emerged as a useful strategy to shed more light on how exposure to different photoperiods impacts physiology and health.

In the study of the seasonal effects, the most used animal models have been long-day breeding rodents, such as hamsters and voles, since they display quick responses to different photoperiods (Krol et al., 2005; Paul et al., 2007). Nevertheless, one of the main drawbacks to using these models is the limited genetic information available in data repositories, in addition to the scarce molecular and biochemical tools to study these seasonal species. For this reason, in recent decades, Fischer 344 rats have become an interesting animal model with which to evaluate the effects of photoperiod exposure since they display a marked physiological and reproductive response to seasonal variations in day length (Heideman and Sylvester, 1997; Heideman et al., 2000; Shoemaker and Heideman, 2002; Yasuo et al., 2007; Ross et al., 2011, 2015; Togo et al., 2012; Tavolaro et al., 2015). As an example, Heideman and Sylvester (1997), Heideman et al. (2000) showed that, after exposure to photoperiods of more than 13.5 h, young Fischer 344 rats displayed higher lean and fat mass, food intake and an increase in testis weight and size compared to those rats exposed to a shorter photoperiod, which exhibited a regressive phenotype. Nevertheless, to the best of our knowledge, the photoperiod effects on glucose and lipid metabolisms, the impairment of which is strongly related to the appearance of risk factors for CVD, including obesity, insulin resistance and dyslipidemia, have not yet been evaluated in this animal model.

In C57BL/6J mice, Tashiro et al. (2017) showed that the exposure to a short photoperiod for 3 weeks produced hyperglycemia, which was partly explained by the down-regulation of the glucose transporter GLUT4 in the gastrocnemius muscle. These authors also demonstrated that C57BL/6J mice held under a short photoperiod displayed increased sucrose intake, body weight and fat mass and a depressive state (Otsuka et al., 2014, 2015), partly resembling subjects suffering from seasonal affective disorder (SAD), a mood disorder in which people exhibit depressive symptoms, hyperphagia, carbohydrate cravings, increased body weight gain and fat accretion, especially in winter (Rosenthal et al., 1984). In humans, it has been shown that a standard oral glucose tolerance test triggers a lower and slower insulin response during spring than during autumn, suggesting seasonal differences in insulin secretion and/or the different blood sugar lowering effects of this hormone at different seasons of the year (Reinberg, 2003). In obese male subjects, it has been observed that the circulating levels of cholesterol, triglycerides and the adipocytokine leptin, which play an important role in long-term regulation of body weight and energy homeostasis, significantly increase during winter (Kanikowska et al., 2014).

All the aforementioned findings prompted us to hypothesize that chronic exposure to different photoperiods would produce changes related with glucose and lipid metabolisms in Fischer 344 rats. Therefore, the main aim of the present study was to determine the effects of a chronic exposition to different photoperiods on glucose, lipid, and energy metabolisms in normoweight Fischer 344 rats. Our goal was accomplished by analyzing different biochemical parameters and key genes and proteins involved in these metabolisms and carrying out an ^1^H NMR metabolomic analysis of the blood, liver and skeletal muscle.

## Materials and Methods

### Animals

The animals used were 8-week-old male Fischer 344 rats (Charles River Laboratories, Barcelona, Spain). After an adaptation period of 4 days, in which animals were housed in pairs at 22°C under a light/dark cycle of 12 h, they were submitted to three light schedules to emulate season’s day length: short day photoperiod (*n* = 6, L6, 6 h light—from Zeitgeber times (ZTs) 0 to 6—and 18 h darkness—from ZTs 6 to 24), normal day photoperiod (*n* = 6, L12, 12 h light—from ZTs 0 to 12—and 12 h darkness—from ZTs 12 to 24) and long day photoperiod (*n* = 6, L18, 18 h light—from ZTs 0 to 18—and 6 h darkness—from ZTs 18 to 24). Rats in each photoperiod were fed *ad libitum* with a standard diet (2.90 kcal⋅g^-1^; Teklad Global 14% Protein Rodent Diet 2014, ENVIGO, Sant Feliu de Codines, Barcelona, Spain). Food intake and body weight data were recorded weekly. After 14 weeks, the animals were deprived of food for 1 h and were sacrificed between ZTs 1 and 2 to minimize the possible circadian variations. The blood was collected, and the serum was obtained by centrifugation and stored at -80°C until analysis. Liver, gastrocnemius and soleus muscle were rapidly removed after death, weighed, frozen in liquid nitrogen and stored at -80°C until further analysis. The Animal Ethics Committee of the University Rovira i Virgili (Tarragona, Spain) approved all the procedures.

### Body Composition Analyses

Lean and fat mass analyses were performed 1 week before the sacrifice using an EchoMRI-700^TM^ device (Echo Medical Systems, L.L.C., Houston, TX, United States). The measurements were performed in duplicate. Data are expressed in absolute (g) and relative values as a percentage of body weight (%). Lean/fat mass ratio was also calculated.

### Serum Analysis

Enzymatic colorimetric assays were used for the analysis of glucose, total cholesterol and triglycerides (QCA, Amposta, Tarragona, Spain), phospholipids (Spinreact, St. Esteve de Bas, Girona, Spain) and non-esterified free fatty acids (NEFAs) (WAKO, Neuss, Germany). Serum insulin and glucagon levels were analyzed using a rat insulin ELISA kit (Millipore, Barcelona, Spain) and a rat glucagon ELISA kit (Cusabio Biotech, Wuhan, China), respectively.

### Total Glycogen Extraction and Quantification

In this method, 750 and 400 mg samples of liver and gastrocnemius muscle, respectively, were boiled for 20 min in a KOH 30% solution. Total glycogen was precipitated by adding saturated Na_2_SO_4_ and 95% ethanol and then centrifuged at 2560 × *g* for 15 min at 4°C. Supernatants were boiled with hydrochloric acid for 2 h to hydrolyze glycogen into glucose and neutralized with sodium chloride. Glucose levels were determined by an enzymatic colorimetric kit (QCA, Amposta, Tarragona, Spain).

### Total Lipid Content Extraction and Quantification

Lipids were extracted from liver (100 mg) and gastrocnemius muscle (200 mg) using the methods described in Hara and Radin (1978) and Rodríguez-Sureda and Peinado-Onsurbe (2005), with the modifications described in Caimari et al. (2012). The quantity of the lipids in both tissues was determined gravimetrically. Both the lipid and aqueous fractions obtained in this extraction were used to perform an NMR analysis for metabolite determination in both tissues.

### Alanine Aminotransferase (ALT) and Lactate Dehydrogenase (LDH) Activity

Fifty milligrams of liver and gastrocnemius muscle were homogenized in 500 μL of ALT or LDH Assay buffer and centrifuged at 10000 × *g* for 15 min at 4°C. ALT and LDH activities were determined using an ALT Activity Assay Kit (Sigma, Madrid, Spain) and LDH Activity Assay Kit (Sigma, Madrid, Spain), respectively.

### Serum Extraction and ^1^H NMR Analysis for Metabolite Determination

Serum metabolites were extracted with methanol:water (8:1). After centrifugation (1800 × *g*, 10 min at 4°C), supernatants containing soluble metabolites were placed into new vials. Pellets resulting from the aqueous extraction were washed twice with methanol:water. Supernatants were dried in an N_2_ stream to remove water and stored at -80°C.

For ^1^H NMR analysis, the aqueous extracts obtained in the serum, liver, and gastrocnemius muscle extractions were reconstituted in 700 μl of a solution containing 0.005% trisilylpropionic acid (TSP) (0.7381 mM) dissolved in D_2_O phosphate buffer (0.05 M). Lipophilic extracts were subsequently dissolved in 700 μl of a solution containing 0.01% tetramethylsilane (TMS) dissolved in CD_3_Cl:CD_3_OD (2:1). Samples were vortexed, homogenized for 5 min and centrifuged (15 min at 14000 × *g*). Finally, the redissolved extractions were transferred into 5 mm NMR glass tubes.

^1^H NMR measurements were performed following the procedure described by Vinaixa et al. (2010).

### NMR Data Analysis

NMR data analysis was performed as previously described (Vinaixa et al., 2010).

### Gene Expression Analysis

Liver, gastrocnemius and soleus muscle total RNA and microRNA were extracted using TriPure reagent (Roche Diagnostic, Sant Cugat del Vallès, Barcelona, Spain) according to the manufacturer’s protocol. To isolate both the total and micro RNA species, samples were incubated overnight with 100% isopropanol at -20°C. The cDNA was synthetized using MuLV reverse transcriptase (Applied Biosystems, Madrid, Spain) and subjected to quantitative PCR amplification using a LightCycler 480 II system with SYBR Green I Master Mix (Roche Diagnostic, Sant Cugat del Vallès, Barcelona, Spain). The reaction was performed according to the instructions provided by the manufacturer. The primers used for the different genes are described in Supplementary Table S1 and were obtained from Biomers.net (Ulm, Germany). The relative expression of each mRNA level was calculated as a percentage of the L12 group, using the -2^ΔΔC_t_^ method with *Ppia*,β*-actin, Hprt*, and *Tfrc* genes as endogenous controls.

### miR Quantitative Real-Time PCR

Soleus muscle miR-194, miR-133, and miR-486 levels were measured using TaqMan Advanced miRNA Assays (Applied Biosystems, Carlsbad, CA, United States). Then, 2.5 ng of RNA was used to synthetize the cDNA using a TaqMan Advanced miRNA cDNA Synthesis Kit (Applied Biosystems, Carlsbad, CA, United States), and it was subjected to quantitative PCR on the LightCycler 480 II system (Roche Diagnostic, Sant Cugat del Vallès, Barcelona, Spain) with SYBR Green PCR Master Mix (Applied Biosystems, Carlsbad, CA, United States). miR-191 was used as an endogenous miRNA.

### Western Blot Analysis

Total and phosphorylated (p) AMP-activated protein kinase (AMPK and (p)-AMPK) (62 kDa) and Akt serine/threonine kinase 2 (Akt2 and (p)-Akt2) (60 kDa) protein levels in the liver, soleus, and gastrocnemius muscle were measured by western blot analysis as previously described (Crescenti et al., 2015) with some modifications. Specifically, the membranes were incubated overnight with the two primary antibodies, mouse anti-Akt2 (L79B2) and rabbit anti-(p)-Akt2 (Ser474) (Cell Signaling, Izasa SA, Barcelona, Spain), diluted 1/2500. Then, the membranes were incubated with goat anti-mouse and goat anti-rabbit secondary antibodies (LI-COR, United States), diluted 1/10000. In the liver samples, β-actin primary antibody was used as an endogenous control (42 kDa) (Abcam, England, United Kingdom). In the soleus and gastrocnemius muscles, α-tubulin was used as a loading control (52 kDa) (Cell Signaling Technology, Barcelona, Spain).

### Statistical Analysis

Data are expressed as the mean ± SEM. Grubbs’ test was used to detect outliers, which were discarded before subsequent analyses. Statistical analyses were performed using SPSS Statistics 22 (SPSS, Inc., Chicago, IL, United States). One-way ANOVA followed by Duncan’s *post hoc* test was used to determine significant differences among the three groups. Student’s *t*-test was used for single statistical comparisons. The level of statistical significance was set at bilateral 5%.

Principal component analysis (PCA) and partial least squares discriminant analysis (PLS-DA) were performed after data normalization and autoscaling using MetaboAnalyst 3.0 software (Xia et al., 2015).

## Results

### The Exposure to Different Photoperiods Altered the Circulating Levels of Glucose and NEFAs

No significant changes among groups were found in cumulative food intake, body weight gain and body composition. Animals exposed to the L6 photoperiod showed residually lower liver weights (*p* = 0.014, Student’s *t*-test) than the L12 group, but no changes were observed in the muscle and testes weights (**Table 1**). The analysis of serum parameters revealed that L6 and L18 animals exhibited significantly higher glucose circulating levels compared to the L12 animals (**Table 1**). Moreover, L6 rats also presented residually higher circulating levels of NEFAS (*p* = 0.031, Student’s *t*-test) than L12 animals (**Table 1**).

**Table 1:** Biometric and serum parameters in rats fed a standard diet and exposed to three different light schedules for 14 weeks.

	L6	L12	L18
**Cumulative food intake (g)**	231 ± 3	230 ± 4	228 ± 5
**Biometric parameters**			
Initial body weight (g)	180 ± 8	201 ± 4	195 ± 10
Final body weight (g)	370 ± 11	381 ± 7	387 ± 13
Body weight gain (g)	191 ± 8	180 ± 7	192 ± 8
Liver (g)	11.86 ± 0.15	12.94 ± 0.30	12.73 ± 0.45
Skeletal muscle (g)	2.08 ± 0.07	2.10 ± 0.04	2.12 ± 0.03
Testes (g)	3.09 ± 0.06	3.02 ± 0.06	3.04 ± 0.04
Fat mass (g)	45.06 ± 1.29	52.72 ± 3.65	55.64 ± 4.41
Fat mass (%)	12.52 ± 0.34	13.93 ± 0.86	14.38 ± 0.75
Lean mass (g)	296 ± 8	308 ± 6	310 ± 9
Lean mass (%)	80.94 ± 1.04	81.48 ± 0.81	80.71 ± 0.67
Lean/fat mass ratio	6.56 ± 0.22	5.98 ± 0.42	5.71 ± 0.37
**Serum parameters**			
Glucose (mmol/L)	7.73 ± 0.19a	6.89 ± 0.09b	7.59 ± 0.19a
Insulin (ng/mL)	4.04 ± 0.66	5.41 ± 0.72	5.54 ± 0.73
Glucagon (ng/mL)	2.66 ± 0.13	2.19 ± 0.29	2.86 ± 0.04
Insulin:glucagon ratio	1.57 ± 0.32	2.69 ± 0.43	1.79 ± 0.24
NEFAs (mmol/L)	0.72 ± 0.06	0.56 ± 0.03	0.62 ± 0.05
Phospholipids (mmol/L)	3.08 ± 0.17	2.64 ± 0.15	2.83 ± 0.10
Triglycerides (mmol/L)	1.60 ± 0.12	1.30 ± 0.12	1.39 ± 0.11
Total cholesterol (mmol/L)	3.26 ± 0.19	2.69 ± 0.19	2.97 ± 0.09

*Male Fischer 344 rats were fed a standard diet and were exposed to three different photoperiods for 14 weeks. Data are expressed as the mean ± SEM (n = 6). One-way ANOVA and Duncan’s post hoc tests were performed to compare the values between the groups and significant differences were represented with different letters (a, b). P, photoperiod effect. The skeletal muscle weight represents the total weight of both soleus and gastrocnemius muscles*.

### Rats Held Under Different Photoperiods Displayed Changes in the Circulating Levels of Nitrogenate Metabolites

By analysis of the serum metabolomics, which were performed using NMR, we found eight nitrogenate metabolites with significant changes among the groups. Creatine, histamine, isoleucine, threonine and tryptophan levels were higher in the L6-photoperiod exposed animals than both the L12 and L18 animals (**Table 2**). This group exhibited higher histidine and tyrosine levels than the L18 group (**Table 2**).

**Table 2:** Concentration of representative serum metabolite concentrations analyzed by nuclear magnetic resonance in response to different photoperiod exposure in animals fed a standard diet for 14 weeks.

Metabolite concentration (μmol/L)	L6	L12	L18
3-Hydroxybutyrate	15.82 ± 1.34a	20.97 ± 2.78ab	27.36 ± 0.99b
Acetate	45.36 ± 2.63a	38.75 ± 1.11b	36.84 ± 0.89b
Alanine	139.29 ± 3.63a	120.06 ± 5.99b	133.98 ± 2.67a
Creatine	81.95 ± 4.84a	65.54 ± 2.09b	58.55 ± 3.96b
Formate	8.25 ± 1.16a	6.99 ± 0.82a	1.65 ± 0.64b
Glutamine	140.65 ± 3.10	127.14 ± 6.28	130.03 ± 4.78
Glycerophosphocholine	55.46 ± 0.88ab	50.97 ± 1.88a	58.43 ± 2.50b
Histamine	5.14 ± 0.50a	2.45 ± 0.33b	2.06 ± 0.35b
Histidine	18.86 ± 0.38a	17.14 ± 0.81a	14.18 ± 0.75b
Isoleucine	19.56 ± 1.12a	15.60 ± 0.70b	14.39 ± 0.73b
Lactate	1193 ± 55a	1357 ± 113ab	1624 ± 134b
Lysine	80.10 ± 0.64	87.41 ± 3.57	77.14 ± 4.90
Oxypurinol	4.23 ± 0.79	2.06 ± 0.17	2.69 ± 0.62
Pyruvate	18.97 ± 0.89a	15.41 ± 1.23b	12.32 ± 0.62c
Threonine	37.03 ± 2.22a	30.01 ± 2.25b	26.37 ± 2.18b
Tryptophan	27.62 ± 0.73a	24.50 ± 0.91b	23.19 ± 0.89b
Tyrosine	18.67 ± 0.55a	17.62 ± 0.58a	15.72 ± 0.50b

*Male Fischer 344 rats were fed a standard diet and were exposed to three different photoperiods for 14 weeks. Data are expressed as the mean ± SEM (n = 6). All the metabolites were obtained by performing a nuclear magnetic resonance (NMR) analysis. One-way ANOVA and Duncan’s post hoc tests were performed to compare the values between groups and significant differences were represented with different letters (a, b, c). P, photoperiod effect.*

The L6 group displayed lower 3-hydroxybutyrate levels than the L18 group and higher acetate levels compared to both the L12 and L18 groups (**Table 2**). All other metabolites that did not reach statistical significance are shown in Supplementary Table S2.

### The Chronic Exposure to Different Photoperiods Modified the Glucose and Glycogen Liver Content and the mRNA Levels of Key Genes Involved in Hepatic Glucose Metabolism

**FIGURE 1:**
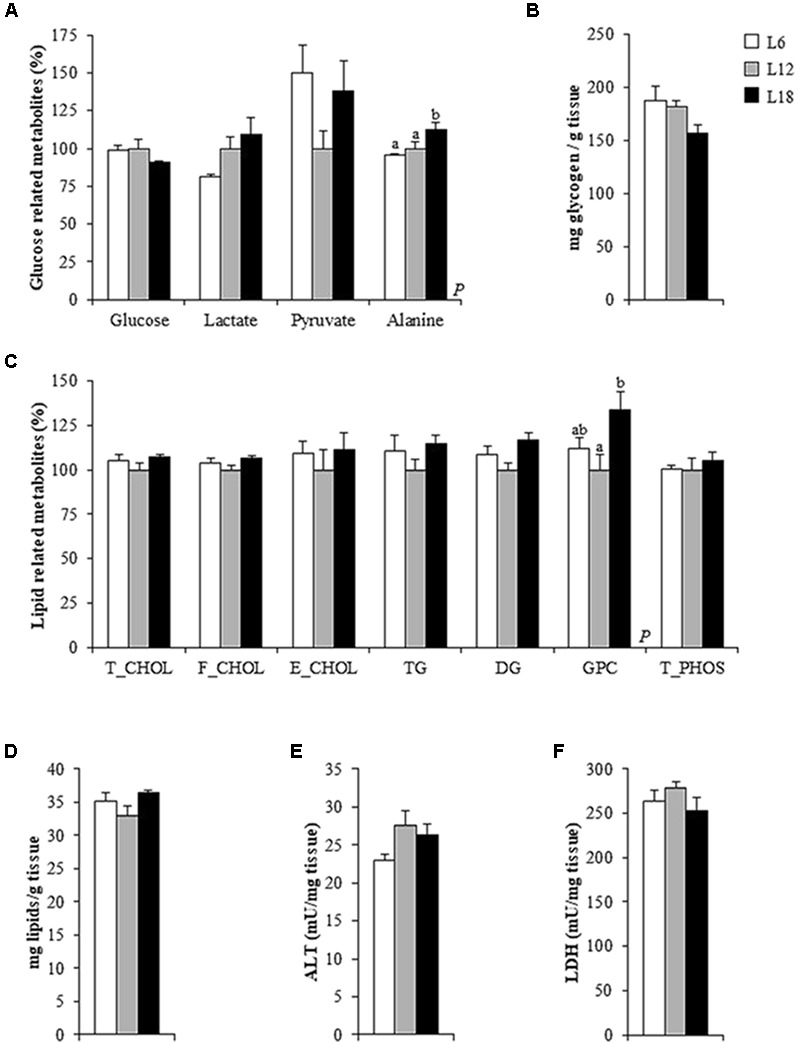
Hepatic glucose **(A)** and lipid-related metabolites **(C)**, glycogen **(B)** and total lipid levels **(D)**, alanine aminotransferase (ALT) **(E)** and lactate dehydrogenase (LDH) activity levels **(F)** in the liver of male Fischer 344 rats fed with a standard diet and exposed to three different photoperiods for 14 weeks. Data are represented as the mean ± SEM (*n* = 6). Liver metabolites concentrations (expressed as μmol/g tissue) are shown in Supplementary Table S3. *P*, photoperiod effect (*p* < 0.05, one-way ANOVA). ^ab^Mean values with different letters are significantly different among the groups (*p* < 0.05, Duncan *post hoc* test). T_CHOL, total cholesterol; F_CHOL, free cholesterol; E_CHOL, esterified cholesterol; TG, triglycerides; DG, diglycerides; GPC, glycerophosphocholine; T_PHOS, total phospholipids.

**FIGURE 2:**
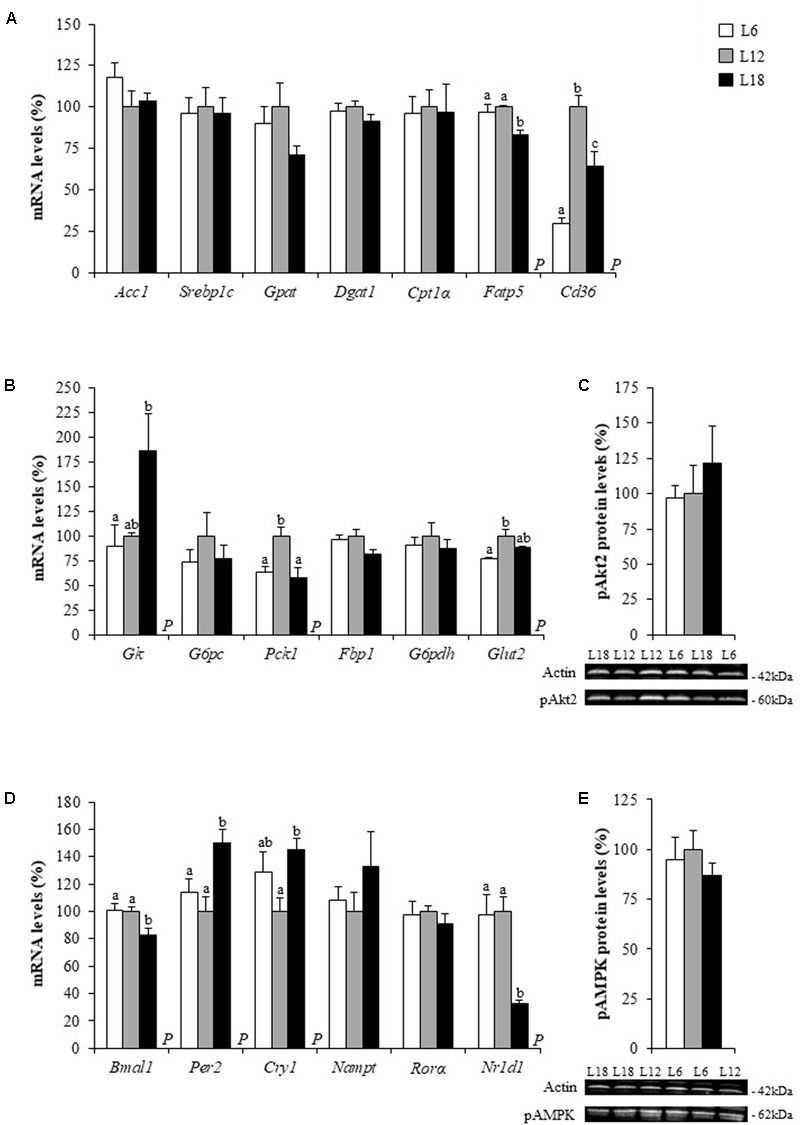
The mRNA levels of lipid metabolism **(A)**, glucose metabolism **(B)** and circadian rhythm-related genes **(D)** and the protein levels of pAkt2 **(C)** and pAMPK **(E)** in the liver of male Fischer 344 rats fed with a standard diet and exposed to three different photoperiods for 14 weeks. Data are represented as the mean ± SEM (*n* = 6). *P*, photoperiod effect (*p* < 0.05, one-way ANOVA). ^ab^Mean values with different letters are significantly different among groups (*p* < 0.05, Duncan *post hoc* test). *Acc1*, acetyl CoA carboxylase 1; β*-actin*, actin beta; *Bmal1*, brain and muscle Arnt-like protein-1; *Cd36*, fatty acid translocase, homolog of CD36; *Cpt1*α, carnitine palmitoyltransferase 1 alpha; *Cry1*, cryptochrome circadian clock 1; *Dgat1*, diacylglycerol acyltransferase 1; *Fatp5*, fatty acid transport protein 5; *Fbp1*, fructose-1,6-biphosphatase 1; *G6pc*, glucose-6-phosphatase, catalytic subunit; *G6pdh*, glucose-6-phosphate dehydrogenase; *Glut2*, glucose transporter 2; *Gk*, glucokinase; *Gpat*, glycerol-3-phosphate acyltransferase; *Hprt*, hypoxanthine guanine phosphoribosyl transferase; *Nampt*, nicotinamide phosphoribosyltransferase; *Nr1d1*, nuclear receptor subfamily 1, group D, member 1; *Per2*, period circadian clock 2; *Pck1*, phosphoenolpyruvate carboxykinase 1; *Ppia*, peptidylprolyl isomerase A; *Rorα*, RAR-related orphan receptor A; *Srebp1c*, sterol regulatory element-binding protein 1c.

To elucidate which mechanisms can be involved in the altered circulating glucose levels observed in both L6 and L18 groups, we measured different glucose metabolism-related parameters in liver, which plays an essential role in glucose homeostasis and is highly regulated by circadian rhythms (Kalsbeek et al., 2014). L18 animals displayed residually lower levels of hepatic glucose than L6 rats (*p* = 0.015, Student’s *t*-test) (**Figure 1A**) and less hepatic glycogen content than the L12 rats (*p* = 0.031, Student’s *t*-test) (**Figure 1B**). The gene expression analyses carried out in the liver revealed that the L18-photoperiod exposed rats displayed a vast overexpression of glucokinase (*Gk*), a key glycolytic-related gene, compared to L6 rats. Moreover, L18 animals displayed lower mRNA levels of the gluconeogenic gene phosphoenolpyruvate carboxykinase 1 (*Pck1*) than L12 rats and residually lower gene expression levels of fructose-1,6-biphosphatase 1 (*Fbp1*) compared to L12 and L6 animals (*p* = 0.05 and *p* = 0.036, Student’s *t*-test, respectively) (**Figure 2B**). L6 animals displayed decreased expression of the genes encoding the glucose transporter 2 (GLUT2) than the L12 animals (**Figure 2B**). All the metabolites obtained by NMR analysis are shown in Supplementary Table S3.

To further explore the mechanisms involved in the photoperiodic regulation of glucose metabolism, the phosphorylated levels of Akt2 and AMPK, two proteins involved in glucose and insulin homeostasis (Cho et al., 2001; Long et al., 2011), were determined in the liver of the three groups of rats. Nevertheless, no changes among the groups were reported in the hepatic levels of these key proteins (**Figures 2C,E**).

### The Lipid Content and Expression of Fatty Acid Transport-Related Genes Changed in the Liver of the Photoperiod Groups

L18 rats displayed significantly greater levels of glycerophosphocholine and residually higher levels of diglycerides (*p* = 0.016, Student’s *t*-test) in this tissue than the L12 group (**Figure 1C**). These metabolic changes observed in L18 rats were accompanied by significant down-regulation of the mRNA levels of the genes codifying the fatty acid transport protein 5 (*Fatp5*) and the fatty acid translocase, homolog of CD36 (*Cd36*) (**Figure 2A**) compared to that in the L12 rats. The *Cd36* mRNA levels were also significantly lower in animals exposed to the L6 photoperiod than in the L12 group animals (70.6% lower) (**Figure 2A**).

### The Exposure to Different Day Lengths Altered the mRNA Levels of Fatty Acid Transport, β-Oxidation and Insulin Signaling-Related Genes and the MicroRNA-194 Expression in the Soleus Muscle

**FIGURE 3:**
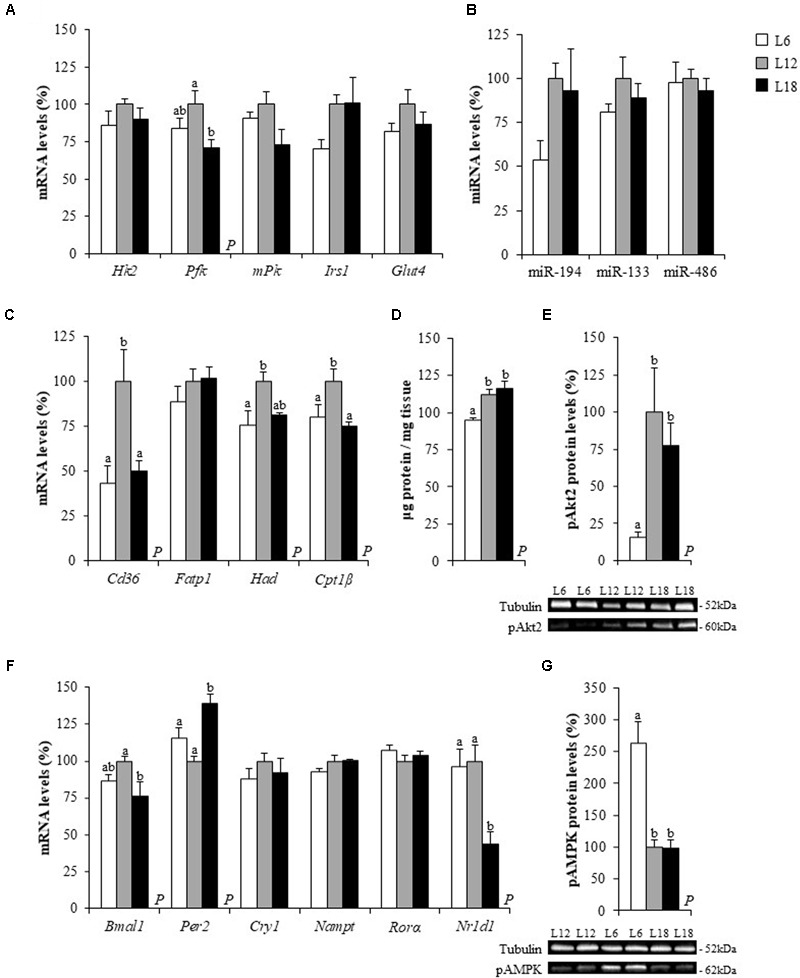
The mRNA levels of glucose metabolism **(A)**, lipid metabolism **(C)** and circadian rhythm-related genes **(F)**, microRNA expression levels **(B)**, the total protein levels **(D)** and the protein levels of pAkt2 **(E)** and pAMPK **(G)** in the soleus muscle of male Fischer 344 rats fed with standard diet and exposed to three different photoperiods for 14 weeks. Data are represented as the mean ± SEM (*n* = 6). *P*, photoperiod effect (*p* < 0.05, one-way ANOVA). ^ab^Mean values with different letters are significantly different among groups (*p* < 0.05, Duncan *post hoc* test). *Cpt1*β, carnitine palmitoyltransferase 1 beta; *Fatp1*, fatty acid transport protein 1; *Glut4*, glucose transporter 4; *Hk2*, hexokinase 2; *Had*, hydroxyacyl-CoA dehydrogenase; *Irs1*, insulin receptor substrate 1; *mPK*, pyruvate kinase type M; *Pfk*, phosphofructokinase; *Tfrc*, transferrin receptor. The rest of the genes analyzed have already been described in **Figure 2**.

**FIGURE 4:**
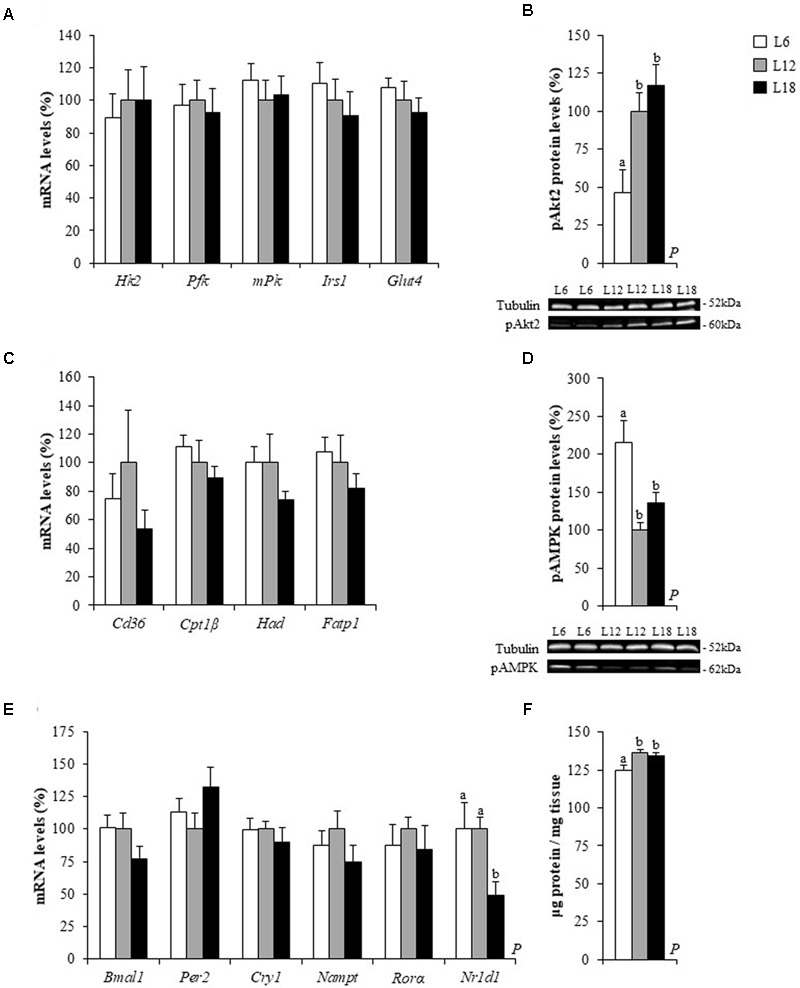
The mRNA levels of glucose metabolism **(A)**, lipid metabolism **(C)** and circadian rhythm-related genes **(E)**, the protein levels of pAkt2 **(B)** and pAMPK **(D)** and the total protein levels **(F)** in the gastrocnemius muscle of male Fischer 344 rats fed with a standard diet and exposed to three different photoperiods for 14 weeks. Data are represented as the mean ± SEM (*n* = 6). *P*, photoperiod effect (*p* < 0.05, one-way ANOVA). ^ab^Mean values with different letters are significantly different among groups (*p* < 0.05, Duncan *post hoc* test). The genes analyzed have been already described in **Figures 2, 3**.

To better characterize the effects of chronic exposure to different photoperiods related to the increase in the circulating levels of glucose and NEFAs, the mRNA levels of a subset of genes involved in fatty acid uptake, β-oxidation, glycolysis and insulin signaling were analyzed in both the soleus and gastrocnemius muscles of the L6, L12, and L18 rats. In the soleus muscle, animals exposed to both short and long photoperiods displayed a significant, sharp down-regulation of the fatty acid transporter *Cd36* mRNA levels (56.5 and 49.8% lower, respectively) compared to L12 rats (**Figure 3C**). Both groups also exhibited lower expression of the β-oxidation-related gene carnitine palmitoyltransferase 1 beta (*Cpt1*β) but only L6 animals displayed significant lower hydroxyacyl-CoA dehydrogenase (*Had*) mRNA levels in comparison with L12 animals (**Figure 3C**). The L18-photoperiod exposed rats showed lower mRNA levels of phosphofructokinase (*Pfk*), a gene involved in the glycolytic process, than the L12 rats (**Figure 3A**). In addition, L6 rats presented lower mRNA levels of the insulin receptor substrate 1 (*Irs1*) (*p* = 0.007, Student’s *t*-test) (**Figure 1A**), a gene encoding a key protein involved in the insulin signaling pathway (Long et al., 2011), and lower expression levels of the glucose metabolism-related microRNA-194 (miR-194) (Latouche et al., 2016) than the L12 rats (*p* = 0.009, Student’s *t*-test) (**Figure 3B**). No significant changes among groups were observed in the expression of these genes in the gastrocnemius muscle (**Figures 4A,C**).

### The NMR Metabolomic Analysis Revealed an Effect of the Photoperiod on the Levels of Lipid and Energy Intermediates in the Gastrocnemius Muscle

**FIGURE 5:**
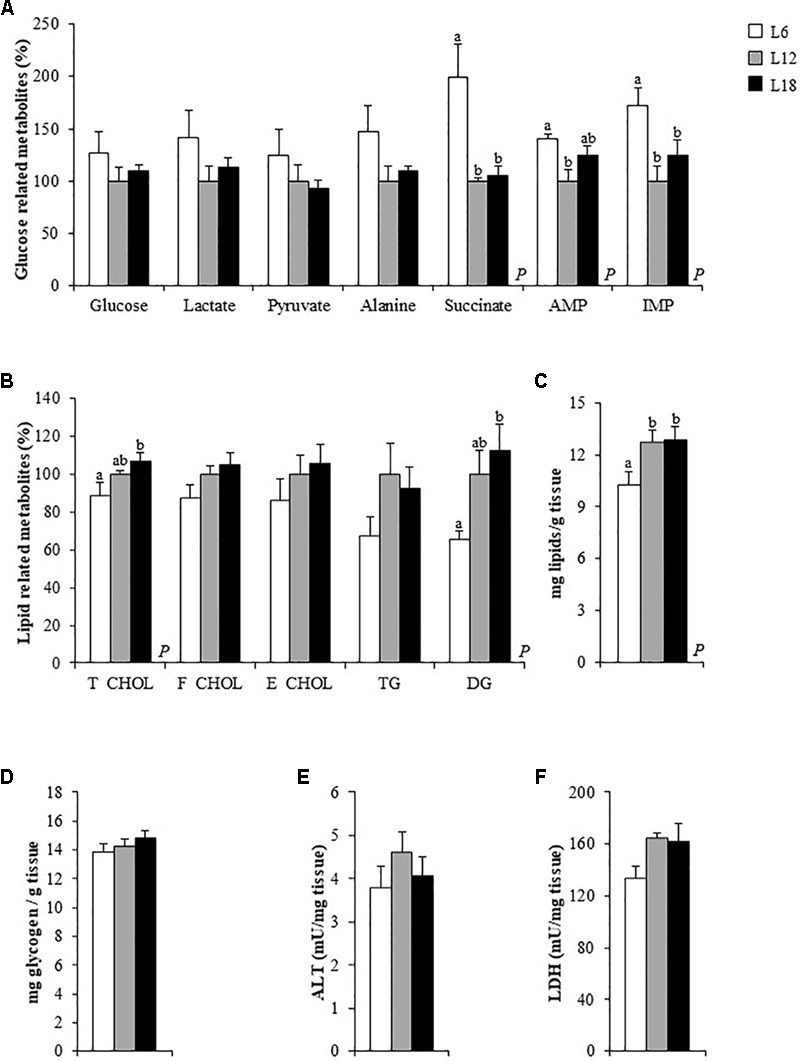
Glucose **(A)** and lipid-related metabolites **(B)**, total lipid **(C)** and glycogen levels **(D)**, alanine aminotransferase (ALT) **(E)** and lactate dehydrogenase (LDH) activity levels **(F)** in the gastrocnemius muscle of male Fischer 344 rats fed a standard diet and exposed to three different photoperiods for 14 weeks. Data are represented as the mean ± SEM (*n* = 6). Gastrocnemius muscle metabolites concentrations (expressed as μmol/g tissue) are shown in the Supplementary Table S4. *P*, photoperiod effect (*p* < 0.05, one-way ANOVA). ^ab^Mean values with different letters are significantly different among groups (*p* < 0.05, Duncan *post hoc* test). AMP, adenosine monophosphate; IMP, inosine monophosphate. The lipid-related metabolites analyzed have been already described in **Figure 1**.

In the gastrocnemius muscle, succinate, AMP and IMP levels were significantly higher in L6 rats compared to the other groups (**Figure 5A**), suggesting an altered energy metabolism caused by exposure to this photoperiod. This group also displayed lower levels of total cholesterol and diglycerides than the L18 group and, consequently, a lower amount of total lipids in this tissue (**Figures 5B,C**). No differences among groups were observed in the muscular glucose and glycogen levels (**Figures 5A,D**). All the metabolites obtained by the NMR analysis are shown in Supplementary Table S4.

### Photoperiod Exposure Slightly Modulated Cori and Cahill Cycles Intermediates in Liver, Skeletal Muscle, and Blood

L6 animals displayed lower serum lactate levels than the L18 animals and higher circulating levels of pyruvate compared to both the L12 and L18 rats (**Table 2**). In addition, L6 animals showed higher hepatic pyruvate levels than L12 animals (*p* = 0.042, Student’s *t*-test) (**Figure 1A**). Both L6 and L18 animals exhibited greater circulating levels of alanine compared to L12 rats (**Table 2**). L18 animals also displayed higher hepatic alanine levels than the rats held under the other photoperiods (**Figure 1A**).

The changes in the levels of pyruvate, alanine and lactate observed among the groups prompted us to evaluate whether exposure to different photoperiods could alter the Cori and Cahill cycles by analyzing the enzymatic activity of ALT and LDH in the muscle and liver. Concerning the Cori cycle, the metabolic pathway in which lactate produced by anaerobic glycolysis in the muscles is released into the bloodstream, transported to the liver and converted to glucose, then returns to the muscles and is metabolized back to lactate (Freminet and Poyart, 1975), only a significant drop in the enzymatic activity of LDH, which catalyzes the conversion of lactate to pyruvate and back, was observed in the gastrocnemius muscle of L6 animals compared to L12 rats (*p* = 0.010, Student’s *t*-test) (**Figure 5F**). Regarding the Cahill cycle, the pathway through which the muscles export pyruvate and amino groups as alanine to the liver, and receive glucose from the liver via the bloodstream (Felig et al., 1970), only a significant decrease in the ALT activity, which, in a reversible manner, converts L-glutamate and pyruvate into α-ketoglutarate and L-alanine, was observed in the liver of the L6 animals compared to L12 rats (*p* = 0.038, Student’s *t*-test) (**Figure 1E**). However, no changes were observed either in the hepatic LDH (**Figure 1F**) or in the muscular ALT (**Figure 5E**) activities. Altogether, these findings would not support a photoperiod effect on the modulation of the Cahill and Cori cycles.

### The Phosphorylated Levels of Akt2 and AMPK Were Photoperiodically Regulated in the Skeletal Muscles

The profound changes triggered by the exposure to different photoperiods in the soleus mRNA levels of genes involved in fatty acid uptake, β-oxidation and insulin signaling as well as in the gastrocnemius AMP and IMP content, prompted us to analyze the phosphorylated levels of Akt2 and AMPK in both muscles to shed more light on the mechanisms that mediated the photoperiodic effects on glucose and lipid metabolisms. In both soleus and gastrocnemius muscles, L6 photoperiod-exposed animals exhibited a sharp down-regulation of the phosphorylated Akt2 levels (p-Akt2) compared to L12 and L18 animals, and this decrease was greater in the soleus muscle (84.5 and 79.9% lower, respectively) than in the gastrocnemius muscle (53.6 and 60.3% lower, respectively) (**Figures 3E, 4B**). Moreover, L6 animals exhibited a vast up-regulation of the phosphorylated levels of AMPK (p-AMPK) compared to L12 and L18 animals in the soleus (163.8 and 167.3% higher, respectively) and gastrocnemius muscle (115 and 57.4% greater, respectively) (**Figures 3G, 4D**). L6 rats also presented a lower total protein content in both the soleus and gastrocnemius muscles than the L12 and L18 animals (**Figures 3D, 4F**).

### The Exposure to the Long Day Photoperiod Altered the Expression of Circadian Rhythm-Related Genes in the Liver and Skeletal Muscles

In addition to affecting seasonal rhythms, it is well known that the light-dark cycle is a key regulator of the daily rhythmicity, which is under the control of an internal circadian clock, which, in turn, plays a very important role in metabolism regulation (Sahar and Sassone-Corsi, 2012; Brown, 2016). Since the alteration of this body clock in both animals and humans exposed to disrupted light:dark cycles results in alterations in glucose and lipid metabolisms (Oishi and Itoh, 2013; Gooley and Chua, 2014; Gooley, 2016), we analyzed the hepatic and muscular mRNA levels of key clock genes to explore whether changes in the expression of these genes could partly explain the metabolic changes observed in rats held under different photoperiods. Animals exposed to the L18 photoperiod displayed lower mRNA levels of the brain and muscle Arnt-like protein-1 (*Bmal1*) gene and higher gene expression levels of its product, period circadian clock 2 (*Per2*), in liver and in the soleus muscle (**Figures 2D, 3F**). Furthermore, this group also showed lower mRNA levels of the nuclear receptor subfamily 1 group D member 1 gene (*Nr1d1*), the repressor of *Bmal1*, in all three tissues (**Figures 2D, 3F, 4E**) compared to its counterparts. Hepatic cryptochrome circadian clock 1 (*Cry1*) gene expression was also greater in L18-photoperiod exposed animals than in those exposed to the L12 photoperiod (**Figure 2D**).

### Multivariate Analysis Allowed the Clear Differentiation of Animals Exposed to Different Photoperiods

**FIGURE 6:**
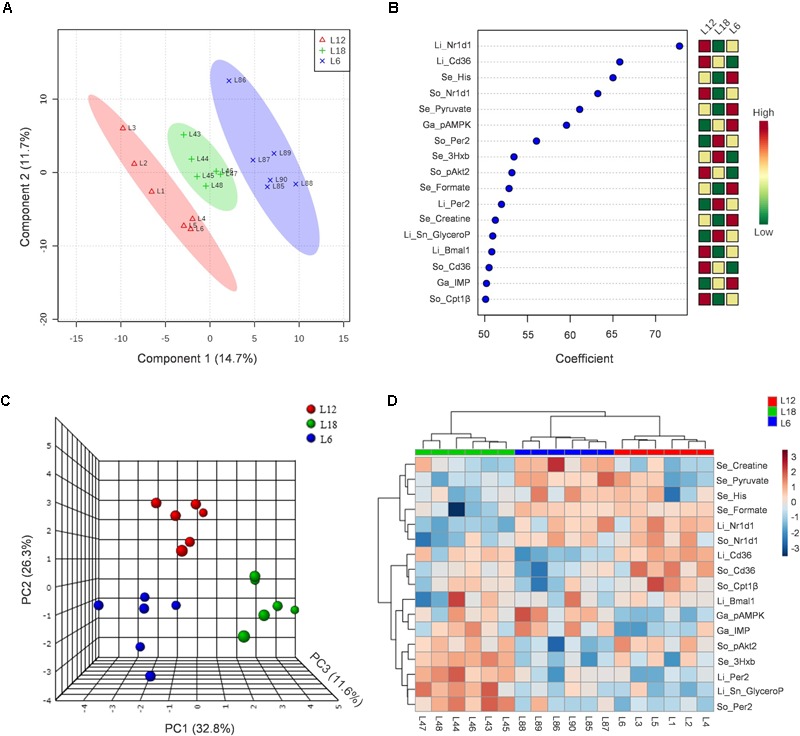
In this study, 239 measured parameters were used to set up a PLS-DA-predictive model **(A)**. Variables with a coefficient mean higher than 50 **(B)** were analyzed in a PCA multivariate analysis **(C)**. The 17 normalized variables were also included in the representation of a Heat Map **(D)**. 3Hxb, 3-hydroxybutyrate; Bmal1, brain and muscle Arnt-like protein-1; Cd36, fatty acid translocase; Cpt1β, carnitine palmitoyltransferase 1 beta; His, histidine; IMP, inosine monophosphate; Nr1d1, nuclear receptor subfamily 1, group D, member 1; Per2, period circadian clock 2; Sn_GlyceroP, Sn-glycerophosphocholine; Ga, gastrocnemius muscle; Li, liver; Se, serum; So, soleus muscle.

First, all 239 parameters measured in this study were analyzed in a PLS-DA predictive model to obtain which were variables with more differences among the three groups (**Figure 6A**). The quality parameters associated with the model were acceptable. When the scores of three components were represented, the degree of fit of the model to the data (*R*^2^) was 0.99, and the result of the cross validation of the model (*Q*^2^) was 0.57, with >0.4 considered an acceptable value for a biological model (Worley and Powers, 2013). Variables with a coefficient mean higher than 50 were selected for PCA multivariate analysis (**Figure 6B**).

A total of 17 variables were obtained and were used to set up a PCA analysis, in which 71% variance was explained when three components were represented. This analysis showed a clear clustering of the different animals depending on the photoperiod in which they were exposed (**Figure 6C**).

Finally, all 17 normalized variables were included in the representation of a heat map, which showed a clear hierarchical clustering among the three groups and revealed which tissues were more affected by each photoperiod (**Figure 6D**).

Among the different parameters that showed higher importance in the separation of the three photoperiod groups, we could mainly observe genes involved in circadian rhythm regulation (*Nr1d1, Per2*, and *Bmal1*) and in fatty acid transport and oxidation (*Cd36* and *Cpt1b*) in both liver and skeletal muscles, as well as some circulating metabolites (pyruvate, histidine, formate, and creatine) and key proteins (pAkt2 and pAMPK) related to glucose and lipid metabolisms and insulin signaling in the skeletal muscle (**Figures 6B,D**).

## Discussion

In the present study, we demonstrated that male Fischer 344 rats exhibited profound changes in parameters related with glucose, lipid and energy metabolisms in the serum, liver and skeletal muscle when chronically exposed to different photoperiods. Thus, the multivariate analysis carried out with the 239 parameters analyzed in this study showed a clear clustering depending on the photoperiod in which the animals were exposed. Unexpectedly, among all these results, no significant changes were obtained in the parameters described as the most affected by exposure to different photoperiods, such as body weight gain, cumulative food intake and testes size (Nelson and Demas, 1997; Prendergast, 2005; Morgan et al., 2006). This lack of changes in the aforementioned parameters could be explained by a possible refractoriness to short days in response to a chronic exposition to fewer hours of light. In this sense, Heideman and Sylvester (1997) and Shoemaker and Heideman (2002) demonstrated that F344 rats could become refractory to the chronic effects of short day photoperiods on body weight (Shoemaker and Heideman, 2002) and testicular parameters (Heideman and Sylvester, 1997; Shoemaker and Heideman, 2002) after 8–10 weeks of exposure. This behavior could be interpreted as an adaptive mechanism to ensure survival and avoid reproductive suppression. Therefore, it is plausible to speculate that, in our study, 14 weeks of exposure to a certain photoperiod would have reversed the photoperiodic effect on these physiological parameters. Another possible reason could rely on the fact that, in the present study, rats were constantly exposed to the same photoperiod for 14 weeks. In nature, the photoperiodic time measurement system is responsive to the direction of the change in day length, in addition to the absolute day length (Prendergast, 2005; Morgan et al., 2006). Therefore, the constant day length exposure that occurred during the experiment could have also dampened the photoperiod effects on those parameters. Interestingly, the refractory response observed in rats exposed to both long and short photoperiods in terms of biometric parameters was not observed in a considerable amount of serum parameters, such as glucose, NEFAs, pyruvate, lactate, and different amino acids (alanine, isoleucine, threonine, histidine, tryptophan, and tyrosine). Remarkably, five out of seventeen parameters that showed a highest contribution to clearly differentiate the three groups in the multivariate analysis were circulating parameters (histidine, pyruvate, 3-hydroxybutyrate, creatine, and formate). These results were also accompanied by clear changes in key genes and proteins involved in both glucose and lipid homeostasis, such as pAkt2, pAMPK, *Cd36*, and *Irs1* levels. Altogether, our findings would indicate that the refractoriness phenomenon observed in biometric parameters were not evident at metabolic and molecular level, suggesting a mismatch in the adaptive responses between biometric and biochemical parameters to this refractory phenomenon.

In this sense, both groups of rats displayed higher circulating glucose levels compared to the L12 group. At first glance, the higher glycaemia observed in both L6 and L18 rats could be tentatively attributed to differences in the feeding state among groups at sacrifice, which could be triggered by a different feeding temporal distribution along light and darkness phases. In this regard, the food intake monitoring during the last 24 h of the study would be useful to shed light on this issue. However, the lack of significant changes in the circulating levels of insulin and glucagon, which are markers of the post-prandial and post-absorptive situations, respectively, as well as the similar insulin:glucagon ratio observed among groups, would strongly suggest that the L6, L12, and L18 animals were at very similar feeding state and that the differences in serum glucose levels were probably explained by other mechanisms. Interestingly, in the L6 animals, the rise of blood glucose levels was accompanied by a sharp down-regulation of the p-Akt2 in both the soleus and gastrocnemius muscles. This protein is considered a crucial mediator of signal transduction processes, playing a key role in apoptosis, cell proliferation, and metabolism regulation (Lawlor and Alessi, 2001; Gonzalez and McGraw, 2009) and is highly expressed in insulin-responsive tissues, such as the liver, skeletal muscle, and adipose tissue (Gonzalez and McGraw, 2009; Yu et al., 2015). After insulin secretion, Akt2 phosphorylation at the Ser^474^ residue promotes the redirection of GLUT4 vesicles from intracellular compartments to the plasma membrane in the skeletal muscle (Cho et al., 2001; Jiang et al., 2003). Considering that ∼80% of the post-prandial glucose uptake occurs in the skeletal muscle (de Lange et al., 2007), it is tempting to hypothesize that the down-regulation of p-Akt2 observed in both skeletal muscles of L6 rats could significantly contribute to the increase in the circulating glucose levels. Furthermore, the down-regulation of the soleus mRNA levels of the gene encoding IRS1, a key protein involved in the activation of Akt2 (Long et al., 2011); the lower levels of miR-194 observed in this tissue, which was also reported in insulin-resistant rats and in prediabetic and diabetic humans (Latouche et al., 2016); and the decreased hepatic gene expression levels of *Glut2*, the main glucose transporter in the liver (Lamia et al., 2008), could also account for the elevated serum glucose levels displayed by L6 rats. Altogether, these results strongly suggest an impairment of glucose homeostasis and insulin signaling in L6 animals, which was mainly demonstrated at the molecular level in the soleus muscle. Our findings partly agree with those obtained by Tashiro et al. (2017), who showed that mice exposed to a short photoperiod over 3 weeks displayed decreased insulin sensitivity. However, these authors described a down-regulation of *Glut4* mRNA and protein levels in the gastrocnemius muscle. This result contrasts with a lack of significant regulation of this gene in our study in both soleus and gastrocnemius muscles. Since the mRNA levels of *Glut4* did not represent an accurate marker of glucose transport in the skeletal muscle (Klip et al., 1994), additional measurements focused on GLUT4 translocation could contribute to clarifying this issue. In addition, the down-regulation of the hepatic gluconeogenic gene *Pck1* observed in L6 rats would neither be in agreement with the hyperglycemia displayed by these animals, since in mice exposed to disrupted daily light-dark cycles this metabolic feature was accompanied by an increase in the mRNA levels of *Pck1* and *G6pc* in liver (Oishi and Itoh, 2013). Further research focused on the hepatic quantification of PCK1 and/or G6PC proteins would be of value to elucidate whether there was an evident alteration of hepatic glucose homeostasis in the animals chronically exposed to the short photoperiod. As it has been fully studied, almost every tissue have molecular clocks that ensure a high robust homeostasis through the rhythmic expression of different metabolic factors (Kalsbeek et al., 2014). In this regard, one limitation of this study is the fact that all the parameters were only analyzed at a single point. Nevertheless, Shavlakadze et al. (2013) have previously described that pAkt protein levels in liver and skeletal muscle did not have periodicity over 24h in *ad libitum* fed mice, differently to what was observed in 24 h-fasted mice, which showed a clear rhythmicity. These results, together with the fact that, in our study, the animals were only deprived of food for 1 h, would suggest that the sharp down-regulation of pAkt2 observed in both soleus and gastrocnemius of L6 animals would not be explained by differences in daily rhythmicity among the three groups and would reinforce our hypothesis pointing toward a decreased insulin sensitivity in these rats.

Another relevant result obtained in our study was that exposure to a short photoperiod produced a sharp upregulation of p-AMPK in the soleus and gastrocnemius muscles compared to that in both the L12 and L18 groups. This finding could be explained, at least in part, by the elevated levels of AMP observed in the gastrocnemius muscle of L6 rats, since this nucleotide is a cellular stress indicator that acts as the main activator of AMPK (Steinberg and Kemp, 2009). In addition, the increase in the gastrocnemius concentration of IMP found in these animals could indirectly contribute to AMPK activation, since IMP can be converted into AMP and, therefore, may increase the AMP/ATP ratio and, consequently, the AMPK activity (Kulkarni et al., 2011). AMPK plays a crucial role in the maintenance of intracellular homeostasis in the skeletal muscle and it is activated in energy-demanding conditions in order to produce some metabolic effects, such as the enhancement of glucose uptake through the stimulation of GLUT4 translocation in the skeletal muscle (Viollet et al., 2007; Wang et al., 2012). Although, at first glance, this result would not support our hypothesis and suggests a decreased glucose uptake by the skeletal muscle in L6 animals, it is important to highlight that rats were sacrificed in post-prandial conditions (after 1 h of fasting). Thus, since the effects of the AMPK pathway on glucose metabolism are mainly produced when cells are metabolically starved, it is plausible to speculate that, in our study, the increased levels of p-AMPK did not significantly contribute to enhancing the glucose uptake in the skeletal muscle. Some studies have demonstrated the presence of a cross-talk between Akt and the AMPK pathways, in which Akt can negatively regulate the AMPK activity (Kovacic et al., 2003; Hahn-Windgassen et al., 2005; Planavila et al., 2005). Hahn-Windgassen et al. (2005) demonstrated that Akt has a crucial role in protein synthesis activating mTOR through direct phosphorylation and inhibition of AMPK-mediated phosphorylation of TSC2, a negative regulator of mTOR. In addition, the important role of Akt2 in the regulation of the skeletal muscle mass and function has also been described, although its main function is related with insulin signaling (Goncalves et al., 2010). These results make it plausible to suggest that exposure to a short photoperiod could induce the activation of the proteolytic process through AMPK activation in both soleus and gastrocnemius muscles. The lower protein content observed in both skeletal muscles and the higher circulating levels of several amino acids, including the non-essential amino acid alanine—involved in the muscular ammonia detoxification (He et al., 2010), would reinforce this idea. Otsuka et al. (2014) demonstrated that exposure to a short photoperiod for 3 weeks significantly increased the plasma levels of many free amino acids and the marker of muscle degradation 3-methylhistidine in C57BL/6J mice, which could also support our hypothesis. However, our results do not point toward an activation of the Cahill cycle, and no changes were observed in the skeletal muscle weight among the groups. Therefore, additional studies focused on the glutamine synthase/glutaminase system in the muscle and liver, as well as the analysis of mTOR protein or ubiquitin-ligases levels, such as MuRF-1 and MAFbx, in the gastrocnemius muscle would be needed to shed more light on this issue (Bond, 2016). The higher circulating levels of amino acids found in L6 animals compared with L12 and L18 animals could also be attributed to changes in amino acid bioavailability or in the use of this molecules as an energy source for the intestinal cells in response to different chronic day length exposure (Bindels and Delzenne, 2013). Another plausible hypothesis that could contribute to explain the increased pAMPK levels observed in the soleus and gastrocnemius muscles of L6 animals would be a higher levels of activity of these rats prior to the sacrifice. Nevertheless, if this were true, and taking into account that AMPK activates lipid catabolic pathways to increase energy production (Carling and Viollet, 2015), the up-regulation of pAMPK would probably have been accompanied by an up-regulation of key genes involved in β-oxidation (*Had, Cpt1*β) as well as in those involved in glucose (*Glut4*) and fatty acid uptake (*Cd36, Fatp1*) in order to deal with a higher energy demanding state. On the contrary, these animals displayed a down-regulation of the mRNA levels of the fatty acid transport-related genes *Cd36* (liver and soleus muscle) and *Fatp5* (liver) which, in turn, could contribute to explain the higher circulating levels of non-esterified free fatty acids observed in L6 rats compared to L12. Jain et al. (2015) demonstrated that Akt2 is critically related to the stimulation of insulin-mediated fatty acid transport. In agreement with these findings, the sharp down-regulation of the phosphorylated Akt2 levels in the soleus muscle of L6 rats could also account for decreased fatty acid transport in the muscle, which, in turn, would produce a decrease in β-oxidation, as demonstrated by the lower mRNA levels of *Had* and *Cpt1*β. Additional research is needed to shed more light on the signaling pathways that could account for the aforementioned hepatic gene expression changes.

Although, in our study, the chronic exposure to 18 h of light also produced clear changes in parameters related with glucose and lipid metabolisms when compared with the exposure to the 12 h light/day photocycle, the molecular mechanisms involved in these effects were not as evident as those reported in response to the shortening of day length. Thus, the increased circulating levels of glucose observed in L18 animals compared to L12 rats cannot be explained by changes in the phosphorylated levels of Akt2 or AMPK in the liver or skeletal muscle. Since it has been described that the accumulation of hepatic lipids—mainly diglycerides—contributes to altered insulin signaling that could trigger a rise in circulating glucose levels (Samuel and Shulman, 2016), it is tempting to speculate that the increased glycerophosphocholine and diglycerides observed in L18 rats could account for this hyperglycemia. On the other hand, the decreased expression of the fatty acid transport-related genes *Fatp5* (liver) and *Cd36* (liver and soleus) and the down-regulation of the soleus *Cpt1*β mRNA levels observed in L18 rats would indicate an alteration of the fatty acid metabolism. However, these molecular changes were not translated into changes in the circulating levels of NEFAs. This response could be tentatively explained by a lower release of these lipids by white adipose tissue—the major contributor of NEFAs to the bloodstream—or by an enhancement of fatty acid uptake by these fat depots or other tissues, which could be understood as a compensatory mechanism addressed to counteract the decreased uptake and utilization of these metabolites in the liver and skeletal muscle (Caimari et al., 2017a,b). Another possible explanation relies on the fact that the gene expression data does not always match protein levels and, therefore, the quantification of the hepatic and muscular levels of these fatty acid metabolism-related proteins would be useful to clarify this issue.

As it has been aforementioned, one of the physiological mechanisms involved in seasonal responsiveness is the synchronization of circannual rhythms to the astronomical season using the photoperiod (Goldman, 2001). In spite of the interest in elucidating whether the clock genes can also act as circannual timers in addition to circadian timers, their complex behavior in different tissues and the influence of other environmental factors makes it difficult to fully understand how they work (Lincoln et al., 2003; Wood and Loudon, 2014). Moreover, it has been described that these clock-related nuclear receptors are involved in the regulation of lipid and glucose metabolism in liver and skeletal muscle and that their dysregulation could produce significant variations in key genes belonging to glucose and lipid metabolic pathways, such as *Gk* and *Cd36* (Lamia et al., 2008; Delezie and Challet, 2011), which were clearly affected by long photoperiod exposure in our study. In this sense, although we could only measure the circadian rhythm-related genes expression at a single point (ZT 1–2), we detected profound changes in *Per2, Bmal1*, and *Nr1d1* in the liver and the soleus and gastrocnemius muscles of L18 animals. These results could contribute to explain the alterations in glucose and lipid metabolism observed in these rats and would suggest that L18 animals displayed a misalignment of the circadian rhythm in comparison with L12 and L6 groups. On the contrary, L6 animals showed a very similar behavior concerning the clock gene expression pattern than L12 rats, suggesting that the metabolic and biochemical changes observed between these two groups were mainly due to the chronic adaptation to different photoperiods. However, a more profound analysis carried out at different daily time points throughout a 24-h period would be needed to corroborate this hypothesis.

## Conclusion

We reported that chronic exposure to short and long day lengths strongly modulates a wide range of parameters related to glucose, lipid, and nitrogenate metabolism in the blood, liver and in both the soleus and gastrocnemius skeletal muscles of normoweight Fischer 344 rats. Furthermore, we have also partly elucidated the molecular mechanisms that would explain the elevated circulating levels of glucose and NEFAs observed in the animals exposed to the short photoperiod, which were as follows: (1) a sharp down-regulation of the phosphorylated Akt2 levels in both soleus and gastrocnemius muscles; and (2) decreased expression in the soleus muscle of the glucose metabolism-related microRNA-194 and lower mRNA levels of the genes involved in glucose metabolism (*Irs1*, soleus, and *Glut2*, liver), β-oxidation (*Had* and *Cpt1*β, soleus) and fatty acid transport (*Cd36*, soleus and liver). Although several studies have demonstrated the effects of circadian rhythm disruption on the development of insulin resistance (Rudic et al., 2004; Marcheva et al., 2010; Harfmann et al., 2016), to the best of our knowledge, this is the first study that showed relevant changes in glucose and lipid metabolism produced by chronic exposition to different photoperiods in Fischer 344 rats. Despite 24-h kinetic analyses of locomotor activity, food intake and clock genes’ expression would have been of great value to strengthen our findings, this study highlight the importance of circannual rhythms in the metabolic homeostasis regulation. In addition, our results pave the way for the use of Fischer 344 rats as a preclinical model to study the effect of the photoperiod on different altered conditions or diseases that occur in humans and are related to lipid and glucose metabolisms, such as obesity, CVD and SAD, which are potentially sensitive to photoperiodic changes (Rosenthal et al., 1984; Pell and Cobbe, 1999; Kanikowska et al., 2014). Further studies carried out with diet-induced obese rats are planned to elucidate whether the exposure to short and long photoperiods exacerbates these metabolic responses under a situation of altered homeostasis robustness.

## Author Contributions

CB, LA, AC, and JdB designed the studies. RM-C, CD-C, AC, and JdB performed the experiments and analyzed the data. RM-C, AC, and LA wrote the manuscript. All authors read, discussed, and approved the final version of the manuscript.

## Conflict of Interest Statement

The authors declare that the research was conducted in the absence of any commercial or financial relationships that could be construed as a potential conflict of interest.
