# Supplementary material for: The Exposure to Different Photoperiods Strongly Modulates the Glucose and Lipid Metabolisms of Normoweight Fischer 344 Rats
Source: Front Physiol. 2018 Apr 19;9:416. doi: 10.3389/fphys.2018.00416 (PMC5917113; doi:10.3389/fphys.2018.00416)
Supplement: Supplementary file 1 [file Table_1.doc]

Supplementary Material

**The exposure to different photoperiods strongly modulates the glucose and lipid metabolisms of normoweight Fischer 344 rats**

**Roger Mariné-Casadó^1^, Cristina Domenech-Coca^2^, Josep Maria del Bas^1^, Cinta Bladé^2^, Lluís Arola^1,2*^, Antoni Caimari^1^**

*** Correspondence:** Prof. Lluís Arola: [lluis.arola@eurecat.org](mailto:lluis.arola@eurecat.org)

**Supplementary Table 1. Nucleotide sequences of primers used for real time quantitative PCR.**

| **Gene** | **Forward primer**  **(5’ to 3’)** | **Reverse primer**  **(5’ to 3’)** | **Tissue** |
| --- | --- | --- | --- |
| *Acc1* | TGCAGGTATCCCCACTCTTC | TTCTGATTCCCTTCCCTCCT | *L* |
| *β-actin* | TACAGCTTCACCACCACAGC | TCTCCAGGGAGGAAGAGGAT | *L* |
| *Bmal1* | GTAGATCAGAGGGCGACGGCTA | CTTGTCTGTAAAACTTGCCTGTGAC | *G, L, S* |
| *Cd36* | GTCCTGGCTGTGTTTGGA | GCTCAAAGATGGCTCCATTG | *G, L, S* |
| *Cpt1α* | GCTCGCACATTACAAGGACAT | TGGACACCACATAGAGGCAG | *L* |
| *Cpt1β* | GCAAACTGGACCGAGAAGAG | CCTTGAAGAAGCGACCTTTG | *G, S* |
| *Cry1* | TGGAAGGTATGCGTGTCCTC | TCCAGGAGAACCTCCTCACG | *G, L, S* |
| *Dgat1* | CAGACAGCGGTTTCAGCAAT | AGGGGTCCTTCAGAAACAGAG | *L* |
| *Fatp1* | TGCTCAAGTTCTGCTCTGGA | CATGCTGTAGGAATGGTGGC | *G, S* |
| *Fatp5* | CCTGCCAAGCTTCGTGCTAAT | GCTCATGTGATAGGATGGCTGG | *L* |
| *Fbp1* | TGACCCTGCCATCAATGAGT | ATGTCTTCATTCCCCGTCGT | *L* |
| *G6pc* | ATTCCGGTGCTTGAATGTCG | TGGAGGCTGGCATTGTAGAT | *L* |
| *G6pdh* | ACCAGGCATTCAAAACGCAT | CAGTCTCAGGGAAGTGTGGT | *L* |
| *Gk* | CTGTGAAAGCGTGTCCACTC | GCCCTCCTCTGATTCGATGA | *L* |
| *Glut2* | AGTCACACCAGCACATACGA | TGGCTTTGATCCTTCCGAGT | *L* |
| *Glut4* | CCATTGCTTCTGGCTATCAC | TCCGTTTCTCATCCTTCAGC | *G, S* |
| *Gpat* | CAGCGTGATTGCTACCTGAA | CTCTCCGTCCTGGTGAGAAG | *L* |
| *Hk2* | GAAGATGCTGCCCACTTACG | GCCATGCATAACCTCCTGTG | *G, S* |
| *Had* | ATCGTGAACCGTCTCTTGGT | AGGACTGGGCTGAAATAAGG | *G, S* |
| *Hprt* | TCCCAGCGTCGTGATTAGTGA | CCTTCATGACATCTCGAGCAAG | *G, L, S* |
| *Irs1* | CTACACCCGAGACGAACACT | TAACCTGCCAGACCTCCTTG | *G, S* |
| *mPk* | AGCCTCCAGTCAATCCACAG | GCATCCTTACACAGCACAGG | *G, S* |
| *Nampt* | CTCTTCACAAGAGACTGCCG | TTCATGGTCTTTCCCCCACG | *G, L, S* |
| *Nr1d1* | ACAGCTGACACCACCCAGATC | CATGGGCATAGGTGAAGATTTCT | *G, L, S* |
| *Per2* | CGGACCTGGCTTCAGTTCAT | AGGATCCAAGAACGGCACAG | *G, L, S* |
| *Pck1* | GCAAACCAGCAAGCACAATG | CTCGAAGTGGAACCAAACCC | *L* |
| *Pfk* | GTGGATGGTGGAGAGCACAT | TCCGATGACACACAGATTGG | *G, S* |
| *Ppia* | CCAAACACAAATGGTTCCCAGT | ATTCCTGGACCCAAAACGCT | *G, L, S* |
| *Rorα* | CCCGATGTCTTCAAATCCTTAGG | TCAGTCAGATGCATAGAACACAAACTC | *G, L, S* |
| *Srebp1c* | CCCACCCCCTTACACACC | GCCTGCGGTCTTCATTGT | *L* |
| *Tfrc* | ATCATCAAGCAGCTGAGCCAG | CTCGCCAGACTTTGCTGAATTT | *S* |

The table shows the nucleotide sequences of primers used for PCR amplification. Primer pairs for PCR were designed using Primer3 software and the sequence information were obtained from Genbank. *Acc1*, acetyl CoA carboxylase 1; *β-actin*, actin beta; *Bmal1,* brain and muscle Arnt-like protein-1; *Cd36*, fatty acid translocase, homologue of CD36; *Cpt1α*, carnitine palmitoyltransferase 1 alpha; *Cpt1β*, carnitine palmitoyltransferase 1 beta; *Cry1,* cryptochrome circadian clock 1; *Dgat1*, diacylglycerol acyltransferase 1; *Fatp1*, fatty acid transport protein 1; *Fatp5*, fatty acid transport protein 5; *Fbp1*, fructose-1,6-biphosphatase 1; *G6pc*, glucose-6-phosphatase, catalytic subunit; *G6pdh*, glucose-6-phosphate dehydrogenase; *Gk*, glucokinase; *Glut2*, glucose transporter 2; *Glut4,* glucose transporter 4; *Gpat*, glycerol-3-phosphate acyltransferase; *Hk2*, hexokinase 2; *Had*, hydroxyacyl-CoA dehydrogenase; *Hprt*, hypoxanthine guanine phosphoribosyl transferase; *Irs1,* insulin receptor substrate 1; *mPK*, pyruvate kinase type M; *Nampt,* nicotinamide phosphoribosyltransferase*; Nr1d1,* nuclear receptor subfamily 1, group D, member 1*; Per2,* period circadian clock 2*; Pck1*, phosphoenolpyruvate carboxykinase 1; *Pfk*, phosphofructokinase; *Ppia*, peptidylprolyl isomerase A; *Rorα*, RAR-related orphan receptor A; *Srebp1c*, sterol regulatory element-binding protein 1c; *Tfrc*, transferrin receptor. Gene expression levels were analyzed in gastrocnemius (G) and soleus (S) muscles and liver (L).
