# Supplementary material for: The Exposure to Different Photoperiods Strongly Modulates the Glucose and Lipid Metabolisms of Normoweight Fischer 344 Rats
Source: Front Physiol. 2018 Apr 19;9:416. doi: 10.3389/fphys.2018.00416 (PMC5917113; doi:10.3389/fphys.2018.00416)
Supplement: Supplementary file 2 [file Table_2.doc]

Supplementary Material

**The exposure to different photoperiods strongly modulates the glucose and lipid metabolisms of normoweight Fischer 344 rats**

**Roger Mariné-Casadó^1^, Cristina Domenech-Coca^2^, Josep Maria del Bas^1^, Cinta Bladé^2^, Lluís Arola^1,2*^, Antoni Caimari^1^**

*** Correspondence:** Prof. Lluís Arola: [lluis.arola@eurecat.org](mailto:lluis.arola@eurecat.org)

# Supplementary Table 2. Concentration of serum metabolite concentrations analysed by Nuclear Magnetic Resonance in response to different photoperiod exposure in animals fed a standard diet for 14 weeks.

| **Metabolite concentration (μmol/L)** | **L6** | **L12** | **L18** |
| --- | --- | --- | --- |
| 2-Methylglutarate | 1.97 ± 0.20 | 2.23 ± 0.08 | 2.40 ± 0.21 |
| Choline | 4.90 ± 0.28 | 4.55 ± 0.17 | 4.77 ± 0.16 |
| Citrate | 11.28 ± 1.18 | 9.40 ± 1.13 | 9.94 ± 0.82 |
| Creatine Phosphate | 5.15 ± 0.13 | 5.35 ± 0.32 | 5.21 ± 0.68 |
| Cytosine | 5.34 ± 0.40 | 4.55 ± 0.44 | 4.87 ± 0.46 |
| Leucine | 32.82 ± 1.32 | 31.66 ± 1.27 | 31.52 ± 1.98 |
| Methionine | 21.34 ± 0.74 | 19.66 ± 1.02 | 20.10 ± 0.88 |
| O-acetylcarnitine | 3.48 ± 0.11 | 3.28 ± 0.24 | 3.18 ± 0.19 |
| Phenylalanine | 10.20 ± 0.34 | 9.59 ± 0.25 | 9.61 ± 0.51 |
| Serine | 34.79 ± 1.43 | 32.70 ± 1.73 | 32.16 ± 1.82 |
| Thymidine | 3.92 ± 0.56 | 4.34 ± 0.37 | 4.26 ± 0.75 |
| Valine | 36.17 ± 1.51 | 34.33 ± 1.11 | 33.02 ± 1.79 |

Male Fischer 344 rats were fed a standard diet and were exposed to three different photoperiods for 14 weeks. Data are expressed as mean ± SEM (n=6). All the metabolites were obtained by performing a Nuclear Magnetic Resonance (NMR) analysis.
