# Supplementary material for: The Exposure to Different Photoperiods Strongly Modulates the Glucose and Lipid Metabolisms of Normoweight Fischer 344 Rats
Source: Front Physiol. 2018 Apr 19;9:416. doi: 10.3389/fphys.2018.00416 (PMC5917113; doi:10.3389/fphys.2018.00416)
Supplement: Supplementary file 3 [file Table_3.doc]

Supplementary Material

**The exposure to different photoperiods strongly modulates the glucose and lipid metabolisms of normoweight Fischer 344 rats**

**Roger Mariné-Casadó^1^, Cristina Domenech-Coca^2^, Josep Maria del Bas^1^, Cinta Bladé^2^, Lluís Arola^1,2*^, Antoni Caimari^1^**

*** Correspondence:** Prof. Lluís Arola: [lluis.arola@eurecat.org](mailto:lluis.arola@eurecat.org)

# Supplementary Table 3. Concentration of liver metabolite concentrations analysed by Nuclear Magnetic Resonance in response to different photoperiod exposure in animals fed a standard diet for 14 weeks.

|  | **L6** | **L12** | **L18** |  |
| --- | --- | --- | --- | --- |
| **Aqueous fraction (µmol/g tissue)** |  |  |  |  |
| 2-Deoxyadenosine | 0.05 ± 0 | 0.04 ± 0.01 | 0.05 ± 0 |  |
| 3-Hydroxybutyrate | 2.41 ± 0.23 | 2.66 ± 0.24 | 2.46 ± 0.31 |  |
| Acetate | 0.23 ± 0.02 | 0.22 ± 0.02 | 0.23 ± 0.03 |  |
| Alanine | 0.91 ± 0.01^a^ | 0.95 ± 0.04^a^ | 1.07 ± 0.04^b^ | *P* |
| Allantoin | 0.22 ± 0.02 | 0.22 ± 0.02 | 0.20 ± 0.02 |  |
| AMP | 0.07 ± 0.01 | 0.07 ± 0.01 | 0.07 ± 0 |  |
| β-Alanine | 0.08 ± 0.01 | 0.07 ± 0.01 | 0.07 ± 0 |  |
| Betaine | 2.93 ± 0.12 | 3.04 ± 0.30 | 3.17 ± 0.09 |  |
| Carnosine | 0.07 ± 0.01 | 0.06 ± 0.01 | 0.08 ± 0 |  |
| Creatine | 0.03 ± 0 | 0.03 ± 0 | 0.03 ± 0 |  |
| Creatinine | 0.02 ± 0 | 0.02 ± 0 | 0.02 ± 0 |  |
| Cytidine | 0.13 ± 0.01 | 0.12 ± 0.01 | 0.13 ± 0.01 |  |
| Dimethylamine | 0.07 ± 0.01 | 0.06 ± 0.01 | 0.05 ± 0 |  |
| Formate | 0.10 ± 0.01 | 0.08 ± 0.01 | 0.09 ± 0.01 |  |
| Glucose | 7.83 ± 0.21 | 7.89 ± 0.48 | 7.15 ± 0.09 |  |
| Glutamate | 0.14 ± 0.01 | 0.14 ± 0.02 | 0.16 ± 0.02 |  |
| Glutamine | 0.50 ± 0.04 | 0.48 ± 0.06 | 0.47 ± 0.01 |  |
| Glycine | 0.58 ± 0.01 | 0.57 ± 0.03 | 0.56 ± 0.01 |  |
| IMP | 0.06 ± 0 | 0.05 ± 0 | 0.05 ± 0.01 |  |
| Inosine | 0.66 ± 0.03 | 0.63 ± 0.03 | 0.69 ± 0.03 |  |
| Isoleucine | 0.10 ± 0 | 0.10 ± 0.01 | 0.10 ± 0.01 |  |
| Lactate | 1.00 ± 0.02 | 1.24 ± 0.10 | 1.35 ± 0.14 |  |
| Leucine | 0.28 ± 0.01 | 0.30 ± 0.03 | 0.27 ± 0.02 |  |
| Mannose | 0.25 ± 0.01 | 0.24 ± 0.02 | 0.22 ± 0.01 |  |
| Methionine | 0.10 ± 0.01 | 0.09 ± 0.01 | 0.10 ± 0.01 |  |
| Niacinamide | 0.38 ± 0.03 | 0.37 ± 0.03 | 0.37 ± 0.02 |  |
| N,N-dimethylglycine | 0.02 ± 0 | 0.02 ± 0 | 0.02 ± 0 |  |
| O-Phosphocholine | 0.07 ± 0 | 0.08 ± 0.02 | 0.06 ± 0 |  |
| Oxypurinol | 0.07 ± 0.01 | 0.07 ± 0.01 | 0.08 ± 0.01 |  |
| Phenylalanine | 0.19 ± 0.01 | 0.19 ± 0.01 | 0.18 ± 0.01 |  |
| Pyruvate | 0.16 ± 0.02 | 0.11 ± 0.01 | 0.15 ± 0.02 |  |
| Sarcosine | 0.04 ± 0 | 0.04 ± 0 | 0.05 ± 0.01 |  |
| Succinate | 0.06 ± 0.01 | 0.08 ± 0.01 | 0.08 ± 0.01 |  |
| Taurine | 2.63 ± 0.13 | 2.57 ± 0.23 | 2.84 ± 0.20 |  |
| Trimethylamine | 0.01 ± 0 | 0.01 ± 0 | 0.01 ± 0 |  |
| Tyrosine | 0.09 ± 0 | 0.09 ± 0 | 0.09 ± 0 |  |
| UDP-Glucoronate | 0.10 ± 0.01 | 0.10 ± 0.01 | 0.10 ± 0.01 |  |
| Uracil | 0.12 ± 0.02 | 0.12 ± 0.02 | 0.11 ± 0 |  |
| Uridine | 0.61 ± 0.02 | 0.58 ± 0.03 | 0.62 ± 0.02 |  |
| Uroconate | 0.19 ± 0.01 | 0.17 ± 0.02 | 0.18 ± 0.01 |  |
| Valine | 0.17 ± 0.01 | 0.18 ± 0.01 | 0.17 ± 0.01 |  |
| Xanthine | 0.09 ± 0.01 | 0.09 ± 0.01 | 0.10 ± 0.01 |  |
| **Lipid fraction (µmol/g tissue)** |  |  |  |  |
| ARA+EPA | 0.02 ± 0 | 0.02 ± 0 | 0.02 ± 0 |  |
| DHA | 0.002 ± 0 | 0.003 ± 0 | 0.003 ± 0 |  |
| Diglycerides | 1.24 ± 0.06 | 1.15 ± 0.04 | 1.34 ± 0.05 |  |
| Esterified cholesterol | 0.61 ± 0.04 | 0.55 ± 0.06 | 0.62 ± 0.05 |  |
| Free cholesterol | 2.78 ± 0.08 | 2.68 ± 0.07 | 2.85 ± 0.04 |  |
| Glycerophosphocholine | 0.04 ± 0^ab^ | 0.04 ± 0^a^ | 0.05 ± 0^b^ | *P* |
| Linoleic acid | 0.02 ± 0 | 0.02 ± 0 | 0.02 ± 0 |  |
| Lysophosphatidylcholine | 2.97 ± 0.08 | 3.25 ± 0.33 | 3.02 ± 0.20 |  |
| Monoglycerides | 0.17 ± 0.01 | 0.17 ± 0.01 | 0.16 ± 0.01 |  |
| MUFA | 0.03 ± 0 | 0.03 ± 0 | 0.03 ± 0 |  |
| Oleic acid | 0.01 ± 0 | 0.01 ± 0 | 0.01 ± 0 |  |
| Omega-3 | 0.01 ± 0 | 0.01 ± 0 | 0.01 ± 0 |  |
| Phosphatidylcholine | 11.23 ± 0.28 | 11.50 ± 0.27 | 12.01 ± 0.36 |  |
| Phosphoethanolamine | 3.82 ± 0.08 | 3.55 ± 0.16 | 3.65 ± 0.14 |  |
| Phosphoinositol | 1.21 ± 0.05 | 1.19 ± 0.10 | 1.27 ± 0.06 |  |
| Plasmalogen | 0.27 ± 0.01 | 0.24 ± 0.01 | 0.27 ± 0.01 |  |
| PUFA | 0.07 ± 0 | 0.07 ± 0 | 0.07 ± 0 |  |
| Sphingomyelin | 0.78 ± 0.02 | 0.74 ± 0.04 | 0.78 ± 0.02 |  |
| Total cholesterol | 3.37 ± 0.11 | 3.21 ± 0.12 | 3.44 ± 0.04 |  |
| Total FA chain | 482.15 ± 21.99 | 456.53 ± 23.86 | 505.93 ± 14.44 |  |
| Total phospholipids | 13.10 ± 0.23 | 13.01 ± 0.82 | 13.72 ± 0.59 |  |
| Triglycerides | 4.66 ± 0.36 | 4.21 ± 0.26 | 4.84 ± 0.18 |  |

Male Fischer 344 rats were fed a standard diet and were exposed to three different photoperiods for 14 weeks. Data are expressed as mean ± SEM (n=6). All the metabolites were obtained by performing a Nuclear Magnetic Resonance (NMR) analysis. One-way ANOVA and Duncan’s post-hoc test were performed to compare the values between groups and significant differences were represented with different letters (a, b). *P* Photoperiod effect.
