# Supplementary material for: The Exposure to Different Photoperiods Strongly Modulates the Glucose and Lipid Metabolisms of Normoweight Fischer 344 Rats
Source: Front Physiol. 2018 Apr 19;9:416. doi: 10.3389/fphys.2018.00416 (PMC5917113; doi:10.3389/fphys.2018.00416)
Supplement: Supplementary file 4 [file Table_4.doc]

Supplementary Material

**The exposure to different photoperiods strongly modulates the glucose and lipid metabolisms of normoweight Fischer 344 rats**

**Roger Mariné-Casadó^1^, Cristina Domenech-Coca^2^, Josep Maria del Bas^1^, Cinta Bladé^2^, Lluís Arola^1,2*^, Antoni Caimari^1^**

*** Correspondence:** Prof. Lluís Arola: [lluis.arola@eurecat.org](mailto:lluis.arola@eurecat.org)

# Supplementary Table 4. Concentration of gastrocnemius muscle metabolite concentrations analysed by Nuclear Magnetic Resonance in response to different photoperiod exposure in animals fed a standard diet for 14 weeks.

|  | **L6** | **L12** | **L18** |  |
| --- | --- | --- | --- | --- |
| **Aqueous fraction (µmol/g tissue)** |  |  |  |  |
| 3-Hydroxybutyrate | 0.13 ± 0.01 | 0.13 ± 0.03 | 0.14 ±0.02 |  |
| 3-Hydroxyisobutyrate | 0.03 ± 0 | 0.02 ± 0 | 0.02 ± 0 |  |
| 3-Methyl-2-oxovalerate | 0.02 ± 0 | 0.01 ± 0 | 0.02 ± 0 |  |
| Acetate | 0.02 ± 0 | 0.02 ± 0 | 0.02 ± 0 |  |
| ADP | 0.43 ± 0.09 | 0.36 ± 0.07 | 0.35 ± 0.07 |  |
| Alanine | 1.66 ± 0.29 | 1.13 ± 0.17 | 1.24 ± 0.05 |  |
| AMP | 0.08 ± 0^a^ | 0.06 ± 0.01^b^ | 0.07 ± 0.01^ab^ | *P* |
| Anserine | 4.01 ± 0.56 | 2.76 ± 0.35 | 2.91 ± 0.12 |  |
| β-Alanine | 0.14 ± 0.02 | 0.10 ± 0.02 | 0.12 ± 0.01 |  |
| Carnitine | 0.58 ± 0.08 | 0.43 ± 0.07 | 0.49 ± 0.02 |  |
| Carnosine | 0.23 ± 0.02 | 0.23 ± 0.01 | 0.23 ± 0.01 |  |
| Choline | 0.04 ± 0 | 0.03 ± 0 | 0.03 ± 0 |  |
| Creatine | 14.50 ± 1.85 | 10.91 ± 1.35 | 12.27 ± 0.41 |  |
| CreatinePhosphate | 0.85 ± 0.09 | 0.68 ± 0.13 | 0.97 ± 0.19 |  |
| Creatinine | 0.21 ± 0.03 | 0.16 ± 0.02 | 0.17 ± 0.01 |  |
| Dimethylglycine | 0.04 ± 0.01 | 0.03 ± 0 | 0.04 ± 0 |  |
| Dimethylsulfone | 0.04 ± 0.01 | 0.03 ± 0 | 0.04 ± 0 |  |
| Fucose | 0.15 ± 0.02 | 0.10 ± 0.01 | 0.13 ± 0.02 |  |
| Fumarate | 0.06 ± 0.01 | 0.06 ± 0.01 | 0.07 ± 0.01 |  |
| Glucose | 1.72 ± 0.28 | 1.36 ± 0.19 | 1.49 ± 0.08 |  |
| Glutamate | 0.36 ± 0.06 | 0.33 ± 0.04 | 0.37 ± 0.03 |  |
| Glutamine | 1.44 ± 0.20 | 1.07 ± 0.16 | 1.29 ± 0.08 |  |
| Glutathione | 0.13 ± 0.03 | 0.09 ± 0.01 | 0.08 ± 0.01 |  |
| Glycerol | 0.32 ± 0.06 | 0.22 ± 0.03 | 0.23 ± 0.01 |  |
| Glycine | 1.23 ± 0.18 | 0.83 ± 0.09 | 0.89 ± 0.06 |  |
| IMP | 0.88 ± 0.09^a^ | 0.51 ± 0.08^b^ | 0.64 ± 0.07^b^ | *P* |
| Inosine+ NAD | 0.27 ± 0.05 | 0.21 ± 0.03 | 0.27 ± 0.02 |  |
| Isoleucine | 0.05 ± 0.01 | 0.04 ± 0.01 | 0.04 ± 0 |  |
| Lactate | 20.67 ± 3.72 | 14.57 ± 2.14 | 16.53 ± 1.31 |  |
| Leucine | 0.11 ± 0.02 | 0.08 ± 0.01 | 0.09 ± 0.01 |  |
| Lysine | 0.12 ± 0.01 | 0.10 ± 0.01 | 0.10 ± 0 |  |
| Methylhistidine | 0.07 ± 0.01 | 0.06 ± 0 | 0.06 ± 0.01 |  |
| NAD+ | 0.08 ± 0.01 | 0.07 ± 0.01 | 0.06 ± 0 |  |
| Niacinamide | 0.17 ± 0.02 | 0.14 ± 0.02 | 0.16 ± 0.01 |  |
| O-Acetylcarnitine | 0.19 ± 0.02 | 0.14 ± 0.01 | 0.16 ± 0.01 |  |
| Panthothenate | 0.01 ± 0 | 0.01 ± 0 | 0.01 ± 0 |  |
| Phenylalanine | 0.09 ± 0.01 | 0.08 ± 0.01 | 0.08 ± 0 |  |
| Proline | 0.29 ± 0.04 | 0.23 ± 0.04 | 0.28 ± 0.02 |  |
| Pyruvate | 0.16 ± 0.03 | 0.13 ± 0.02 | 0.12 ± 0.01 |  |
| Succinate | 0.20 ± 0.03^a^ | 0.10 ± 0^b^ | 0.11 ± 0.01^b^ | *P* |
| Taurine | 6.37 ± 0.79 | 5.06 ± 0.61 | 6.12 ± 0.41 |  |
| Tyrosine | 0.09 ± 0.01 | 0.07 ± 0.01 | 0.07 ± 0 |  |
| Valine | 0.10 ± 0.02 | 0.08 ± 0.01 | 0.09 ± 0.01 |  |
| Xanthine | 0.05 ± 0.01 | 0.05 ± 0 | 0.05 ± 0 |  |
| **Lipid fraction (µmol/g tissue)** |  |  |  |  |
| ARA+EPA | 0.02 ± 0 | 0.01 ± 0 | 0.01 ± 0 |  |
| DHA | 0.01 ± 0 | 0.01 ± 0 | 0.01 ± 0 |  |
| Diglycerides | 3.82 ± 0.56 | 5.67 ± 0.92 | 5.22 ± 0.66 |  |
| Esterified cholesterol | 0.17 ± 0.02 | 0.20 ± 0.02 | 0.21 ± 0.02 |  |
| Free cholesterol | 0.84 ± 0.07 | 0.96 ± 0.04 | 1.00 ± 0.06 |  |
| Linoleic acid | 0.03 ± 0 | 0.03 ± 0 | 0.03 ± 0 |  |
| Monoglycerides | 0.07 ± 0.01 | 0.05 ± 0.01 | 0.06 ±0.01 |  |
| MUFA | 0.04 ± 0 | 0.04 ± 0 | 0.04 ± 0 |  |
| Oleic acid | 0.01 ± 0 | 0.02 ± 0 | 0.02 ± 0 |  |
| Omega-3 | 0.02 ± 0 | 0.01 ± 0 | 0.02 ± 0 |  |
| Phosphatidylcholine | 4.22 ± 0.36 | 4.78 ± 0.14 | 4.94 ± 0.35 |  |
| Phosphoethanolamine | 1.97 ± 0.23 | 2.22 ± 0.10 | 2.42 ± 0.22 |  |
| Phosphoinositol | 0.40 ± 0.04 | 0.45 ± 0.02 | 0.48 ± 0.03 |  |
| Plasmalogen | 0.63 ± 0.11 | 0.75 ± 0.02 | 0.85 ± 0.11 |  |
| PUFA | 0.11 ± 0.01 | 0.10 ± 0.01 | 0.10 ± 0.01 |  |
| Sphingomyelin | 0.11 ± 0.01 | 0.13 ± 0 | 0.13 ± 0.01 |  |
| Total cholesterol | 0.94 ± 0.07^a^ | 1.06 ± 0.02^ab^ | 1.13 ± 0.04^b^ | *P* |
| Total FA chain | 123.45 ± 12.73 | 167.15 ± 16.91 | 162.07 ± 14.78 |  |
| Triglycerides | 3.82 ± 0.56 | 5.67 ± 0.92 | 5.22 ± 0.66 |  |

Male Fischer 344 rats were fed a standard diet and were exposed to three different photoperiods for 14 weeks. Data are expressed as mean ± SEM (n=6). All the metabolites were obtained by performing a Nuclear Magnetic Resonance (NMR) analysis. One-way ANOVA and Duncan’s post-hoc test were performed to compare the values between groups and significant differences were represented with different letters (a, b). *P* Photoperiod effect.
